# Artificial intelligence in glioma research: a bibliometric analysis of global trends, hotspots, and future directions

**DOI:** 10.3389/fneur.2025.1701499

**Published:** 2026-01-12

**Authors:** Da Shao, Shulei Lou, Yunsheng Liu, Zengwei Kou

**Affiliations:** 1 Research Center of Translational Medicine, School of Medicine, Shanghai Children’s Hospital, Shanghai Jiao Tong University, Shanghai, China; 2 Linyi People’s Hospital, Linyi, China; 3 Cancer Center, Shenzhen Hospital (Futian) of Guangzhou University of Chinese Medicine, Shenzhen, China; 4 Department of Laboratory Medicine and Pathobiology, Temerty Faculty of Medicine, University of Toronto, Toronto, ON, Canada

**Keywords:** gliomas, artificial intelligence, bibliometrics, machine learning, tumor microenvironment, neuro-oncology

## Abstract

**Background:**

Gliomas are the most common malignant primary brain tumors in adults and remain one of the greatest therapeutic challenges due to their infiltrative growth, molecular heterogeneity, and poor prognosis. With the rapid development of artificial intelligence (AI), increasing efforts have been made to apply AI tools across different stages of glioma research and clinical care.

**Objective:**

This study aims to provide a comprehensive bibliometric analysis of global research activity at the intersection of AI and gliomas, identifying leading contributors, emerging hotspots, and temporal trends.

**Methods:**

We systematically identified relevant publications from the past decade (January 2016 to June 2025) through searches of Web of Science, PubMed, and Scopus. The retrieved records underwent a rigorous de-duplication process and manual validation to ensure data integrity. Key bibliometric indicators were then extracted and analyzed using CiteSpace, Bibliometrix, and VOSviewer to evaluate publication growth trajectories, contributions by countries and institutions, journal co-citation networks, author influence, keyword evolution, and emerging research frontiers.

**Results:**

A total of 16,656 unique publications were identified, exceeding earlier bibliometric estimates. The cumulative number of publications exhibited exponential growth (*R*^2^ = 0.99). China emerged as the most productive country, with significant contributions from leading institutions worldwide. Co-citation and keyword analyses revealed strong clustering around research themes such as tumor microenvironment, molecular profiling, machine learning, radiomics, and microphysiological systems, reflecting the expanding role of AI in precision diagnosis, prognostication, and treatment optimization.

**Conclusion:**

AI has become increasingly integrated into glioma research, complementing traditional diagnostic and therapeutic strategies. By highlighting global research patterns and emerging topics, this study provides valuable insights into the evolving landscape of AI applications in gliomas and suggests future directions for clinical translation.

## Introduction

Gliomas are the most common primary malignant brain tumors in adults, accounting for approximately 30–40% of all central nervous system (CNS) tumors (1). They are characterized by their infiltrative growth into surrounding brain tissue, which complicates both diagnosis and treatment (2). The typical clinical manifestations include signs of intracranial hypertension, neurological and cognitive impairments, and epileptic seizures. Globally, the annual incidence of gliomas is estimated at 5–6 cases per 100,000 population, with geographic and age-related variations (3).

Gliomas are highly heterogeneous, encompassing diverse histological types and genetic alterations that confer variable prognoses. According to the 2021 fifth edition of the WHO Classification of Tumors of the Central Nervous System, gliomas now include multiple distinct astrocytic, oligodendroglial, and glioneuronal entities, with classification increasingly dependent on integrated histological and molecular profiling (2). Dozens of genes, including IDH1/2, ATRX, TP53, and 1p/19q codeletion, have been implicated in gliomagenesis and are now essential for precise diagnosis and prognostication (4). High-grade gliomas such as glioblastoma carry a median survival of less than 2 years despite aggressive multimodal treatment (3), whereas lower-grade variants such as oligodendrogliomas or IDH-mutant astrocytomas may confer longer survival but remain challenging in terms of differential diagnosis, tumor sampling, and pathological confirmation (5).

From a clinical perspective, glioma management requires multidisciplinary integration. Surgical resection remains the cornerstone of therapy to relieve mass effect and intracranial pressure (6), but its safety and efficacy are strongly dependent on preoperative neuroimaging, intraoperative navigation, functional mapping, and neurosurgical expertise. Adjuvant radiotherapy and chemotherapy (e.g., temozolomide) are critical, with ongoing refinements in dose optimization and timing. Meanwhile, systemic and supportive therapies, as well as novel approaches including molecularly targeted agents and immunotherapies, are under active clinical investigation (7). However, CNS-directed therapies often cause collateral neurological injury, resulting in post-treatment deficits in physical and psychological function that necessitate structured rehabilitation and long-term supportive care (8).

In recent years, artificial intelligence (AI) has emerged as a transformative tool in glioma research and clinical care (9, 10). AI-based methods have been applied across the continuum of glioma management: from epidemiological modeling and molecular subtype prediction to radiological diagnosis and automated segmentation, personalized treatment optimization, outcome prediction, and rehabilitation support (11–13). These approaches have demonstrated potential to improve diagnostic accuracy, reduce inter-observer variability, and assist in precision oncology.

To better understand the trajectory of AI in glioma research, we conducted a systematic bibliometric study using three major biomedical databases-Web of Science, PubMed, and Scopus-to identify publications at the intersection of AI and gliomas. We analyzed key contributors (countries, institutions, and authors), influential journals, emerging research hotspots, and temporal trends. In particular, we emphasize how AI-assisted methods intersect with traditional glioma management strategies, highlighting both current advancements and future opportunities.

## Methods and materials

### Literature search strategy

As previously described (14–17), we conducted a systematic literature search from the past decade (January 2016 to June 2025) across PubMed, Web of Science (WoS) core collection, and Scopus, which are internationally recognized as the most comprehensive databases for biomedical and clinical research. Boolean operators were applied to combine synonyms of gliomas and AI, with database-specific adjustments to account for indexing differences.

*PubMed*: (“Artificial Intelligence”[Mesh] OR “Machine Learning”[Mesh] OR “Deep Learning”[Mesh] OR “Neural Networks, Computer”[Mesh] OR “Natural Language Processing”[Mesh] OR artificial intelligence[tiab] OR machine learning[tiab] OR deep learning[tiab] OR neural network*[tiab] OR “convolutional neural network*”[tiab] OR CNN[tiab] OR transformer*[tiab] OR “support vector machine*”[tiab] OR SVM[tiab] OR “random forest*”[tiab] OR “gradient boosting”[tiab] OR xgboost[tiab] OR “natural language processing”[tiab] OR NLP[tiab] OR “computer vision”[tiab] OR radiomic*[tiab] OR radiogenomic*[tiab] OR “computer-aided diagnos*”[tiab] OR CAD[tiab] OR “deep neural network*”[tiab]) AND (“Glioma”[Mesh] OR “Glioblastoma”[Mesh] OR glioma*[tiab] OR glioblastoma*[tiab] OR GBM[tiab] OR astrocytoma*[tiab] OR oligodendroglioma*[tiab] OR ependymoma*[tiab] OR “diffuse midline glioma*”[tiab] OR “brainstem glioma*”[tiab] OR “brain stem glioma*”[tiab] OR “midline glioma*”[tiab]).

*Web of science (WoS)*: TS = (“artificial intelligence” OR “machine learning” OR “deep learning” OR “neural network*” OR “convolutional neural network*” OR CNN OR transformer* OR “support vector machine*” OR SVM OR “random forest*” OR “gradient boosting” OR xgboost OR “natural language processing” OR NLP OR “computer vision” OR radiomic* OR radiogenomic* OR “computer-aided diagnos*” OR CAD OR “deep neural network*”) AND TS = (glioma* OR glioblastoma* OR GBM OR astrocytoma* OR oligodendroglioma* OR ependymoma* OR “diffuse midline glioma*” OR “brainstem glioma*” OR “brain stem glioma*” OR “midline glioma*”).

*Scopus*: TITLE-ABS-KEY(“artificial intelligence” OR “machine learning” OR “deep learning” OR “neural network*” OR “convolutional neural network*” OR CNN OR transformer* OR “support vector machine*” OR SVM OR “random forest*” OR “gradient boosting” OR xgboost OR “natural language processing” OR NLP OR “computer vision” OR radiomic* OR radiogenomic* OR “computer-aided diagnos*” OR CAD OR “deep neural network*”) AND TITLE-ABS-KEY(glioma* OR glioblastoma* OR GBM OR astrocytoma* OR oligodendroglioma* OR ependymoma* OR “diffuse midline glioma*” OR “brainstem glioma*” OR “brain stem glioma*” OR “midline glioma*”).

### Data extraction and processing

Data extraction and processing were performed as follows: records were downloaded in their native formats (.nbib from PubMed, .txt from Web of Science, and .csv from Scopus). To ensure consistency, PubMed and Scopus files were converted to the .txt format, achieving a 100% conversion success rate for PubMed and greater than 99% for Scopus. The resulting results were pre-processed and converted into CiteSpace (6.4. R1) (18). Duplicate detection was performed in CiteSpace, which identifies duplicates based on the following fields: first author’s last name, the first 10 letters of the title, the first 10 letters of the source (journal), year of publication, DOI (if available), and first page number. After deduplication, PubMed retained 1,151 articles, Scopus retained 8,465, and Web of Science Citation Index retained 7,183 (Supplementary Figure 1). The merged dataset was imported into CiteSpace, VOSviewer (1.6.20) (19), and Bibliometrix (5.1.1) (20) for in-depth analysis, enabling the visualization and exploration of research trends, hotspots, and key contributions.

Specific analyses were implemented with the following approaches: publication output-including total and annual trends by document type, country, institution, journal, and author-along with AI keyword trends, were analyzed using R scripts integrating Bibliometrix and other R packages. Global distribution of publications by country, the relationship between national output and citations, institutional productivity, inter-journal citation relationships, and three-field plots were also generated using Bibliometrix in combination with other R packages. Keyword clouds and co-occurrence plots depicting AI- and glioma research-related keyword relationships were produced similarly. Citation networks among countries, institutions, journals, and authors were visualized with VOSViewer. CiteSpace was applied to identify top 20 entities with the strongest citation bursts (institutions and references) and top 20 most cited journals. The operations involving CiteSpace and VOS Viewer primarily utilize default parameters. Bibliometrix, however, employs a customized R script. We have uploaded all source files, intermediate files, and the relevant R script to Zenodo (10.5281/zenodo.17698407).

For additional visualizations, such as publication growth curves and statistical comparisons, GraphPad Prism 8 was used. Comparative analyses between two groups were performed using Student’s two-tailed *t*-test, with *p*-values reported accordingly.

## Results

### Publication trends and document types

**Figure 1:**
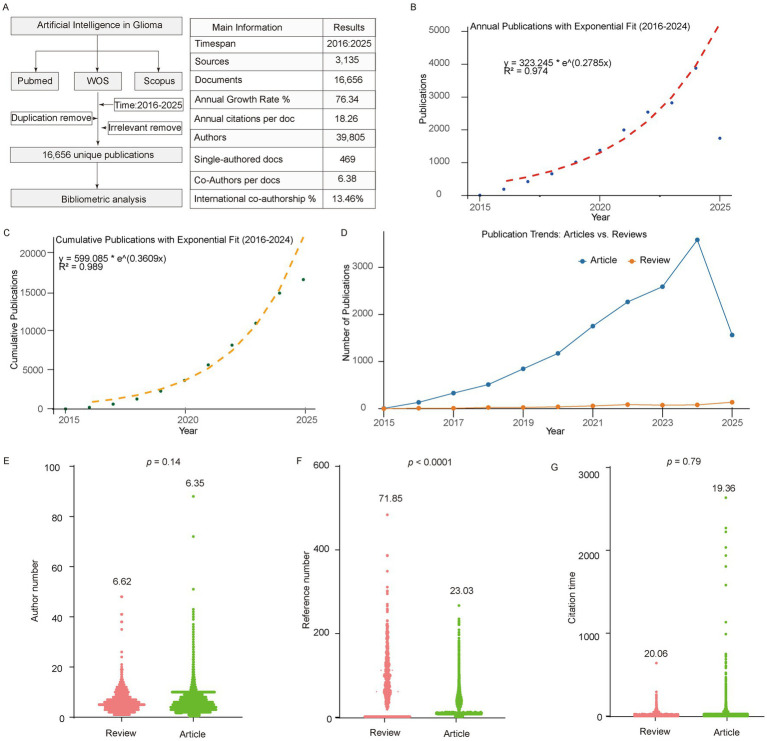
Basic information of the retrieved records. **(A)** Overview of search strategy and main bibliometric information. **(B)** Annual number of publications over time. **(C)** Cumulative publications over time fitted with an exponential model. **(D)** Distribution of document types (articles vs. reviews). **(E–G)** Comparison between articles and reviews in terms of number of authors, references, and citation counts.

According to our search strategy, a total of 4,509 records were retrieved from PubMed, 7,183 from Web of Science, and 10,517 from Scopus (from January 2016 to August 2025). After de-duplication, 16,656 unique publications were included, corresponding to an overall annual growth rate of 76.34%. Most of these publications were co-authored, with single-authored works accounting for less than 3%, and the average number of authors per paper was 6.38 (Figure 1A). The number of annual publications and cumulative publications both showed a rapid increase, fitting an exponential growth model with coefficients of determination (R^2^) of 0.97 and 0.99, respectively (Figures 1B,C). In terms of document types, the majority of publications were original research articles, whereas reviews accounted for only a small proportion (Figure 1D). Notably, reviews had significantly higher numbers of references compared with original articles, but there were no significant differences between the two in terms of number of authors or citation counts (Figures 1E–G).

### High-impact countries and institutions

**Figure 2:**
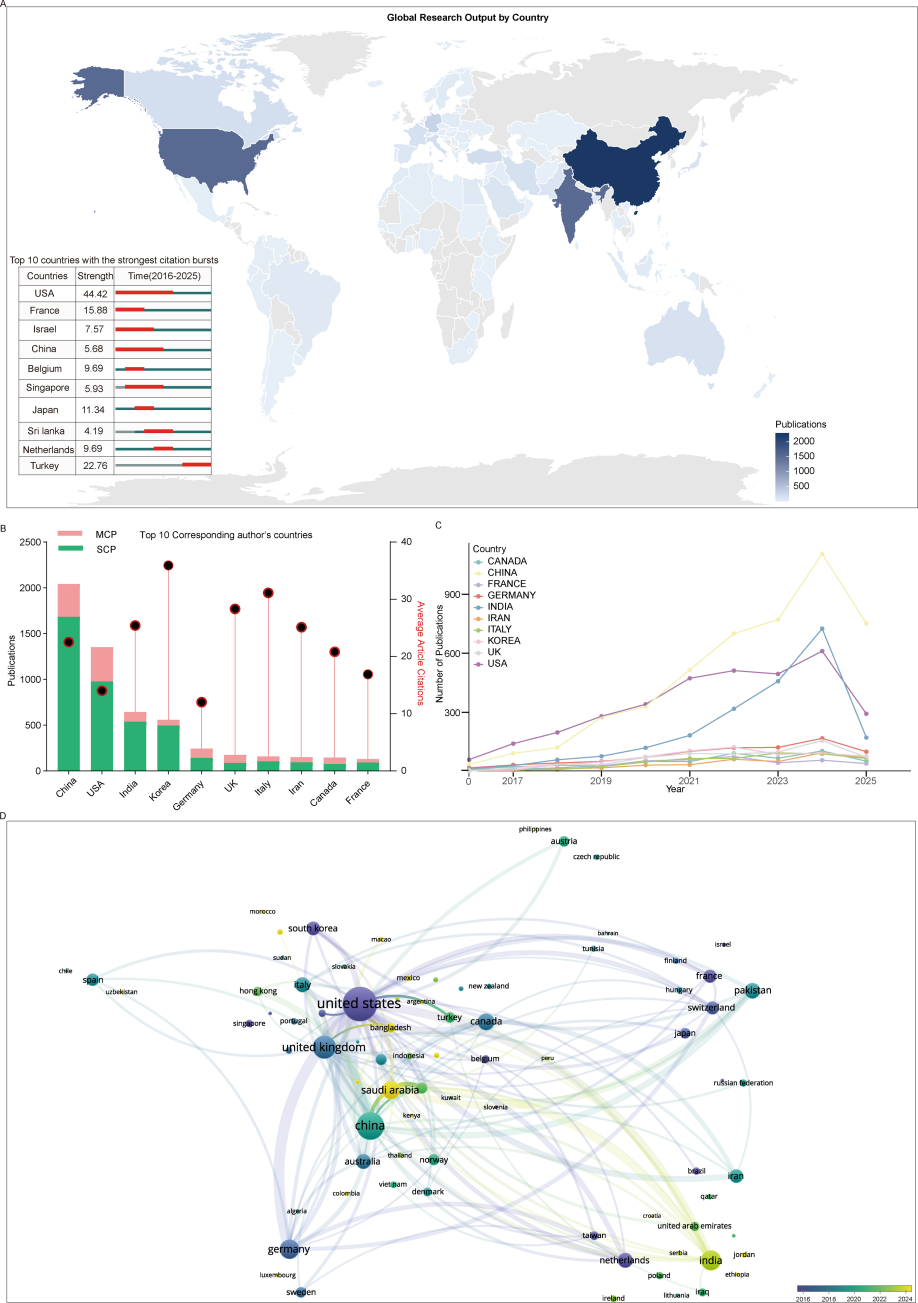
Country-level publication trends and collaborations. **(A)** World map illustrating publication volumes, with inset showing the top 10 countries with the strongest citation bursts. **(B)** Top 10 corresponding author countries ranked by publication volume. **(C)** Publication trends of the top 10 countries over time. **(D)** Co-authorship network of countries, where node size indicates publication volume and node color indicates active period.

China and the United States emerged as the two most productive countries, with 2,042 and 1,354 publications corresponding to the country of the corresponding author, respectively (Figures 2A,B). Other leading countries included India, Korea, and Germany. Interestingly, Korea achieved the highest average citation per paper, more than twice that of Germany, which ranked at the bottom among the top 10. In terms of citation bursts, the United States demonstrated the strongest citation burst (44.42), far surpassing Sri Lanka (4.19) and China (5.68), both of which were also included in the top 10 list (Figures 2A,B).

We observed that the United States consistently led in annual publication output until 2020. Subsequently, China demonstrated a marked increase, surpassing the United States in 2021, with the gap continuing to widen (Figure 2C). Another notable trend was the rapid growth in India’s publication output beginning in 2019. By 2023, India’s output had reached a level comparable to that of the United States and exceeded it in 2024. The growth trends of other countries generally paralleled that of the United States, albeit on a smaller scale.

Analysis of international collaboration networks reveals that several high-productivity countries have formed distinct collaborative hubs. The most prominent hubs center around the United States, China, the United Kingdom, and India. The United States maintains strong collaborative ties with China, Germany, the United Kingdom, and Pakistan, among others. China’s primary collaborators include the United Kingdom, Pakistan, South Korea, Germany, and India. Meanwhile, the United Kingdom’s collaboration network is predominantly concentrated within Europe, with Germany, Italy, and the Netherlands as its main partners (Figure 2D).

From a temporal perspective, newer research hubs have emerged, notably India, Saudi Arabia, and Bangladesh. India has established substantial collaborative relationships with the United States, China, Australia, and South Korea. Interestingly, Saudi Arabia’s strongest collaborative partnership is with India, reflecting shifting patterns in global research cooperation.

**Figure 3:**
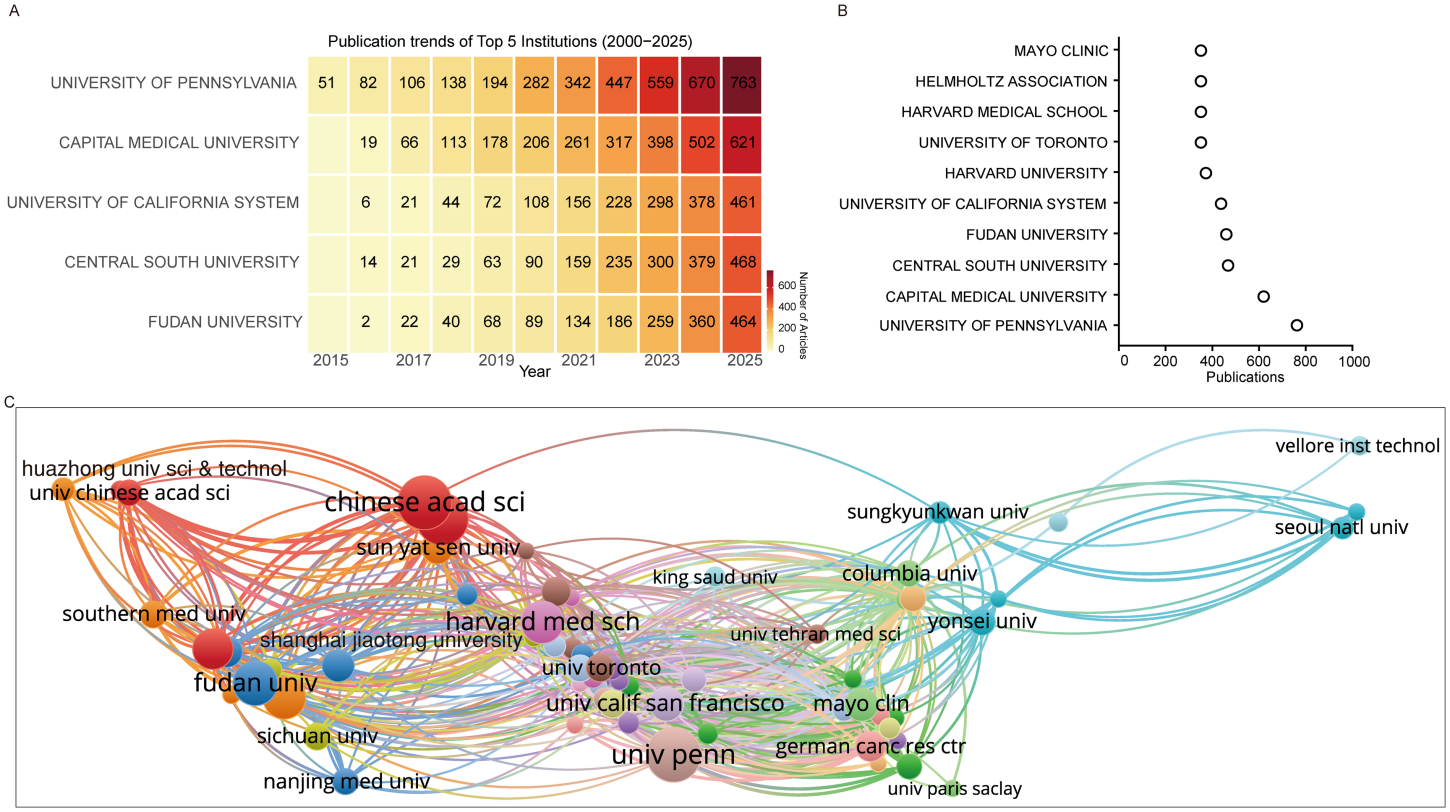
High-impact institutions. **(A)** Publication trends of the top five institutions. **(B)** Top 10 institutions by cumulative publication output. **(C)** Collaboration networks between institutions.

At the institutional level, the University of Pennsylvania ranked first in cumulative publications, followed by Capital Medical University, the University of California System, Central South University, and Fudan University. Institutions ranked 6–10 included several leading North American universities such as Harvard University and the University of Toronto (Figures 3A,B). Similar to the national-level trend, institutional output exhibited accelerated growth after 2020. Collaboration networks revealed a distinct geographical pattern: Chinese institutions such as the Chinese Academy of Sciences collaborated predominantly with domestic partners including Fudan University and Southern Medical University, whereas North American institutions such as Harvard Medical School formed strong ties with the University of Pennsylvania, University of Toronto, and University of California, San Francisco. Korean universities such as Yonsei University, Sungkyunkwan University, and Seoul National University also formed closely connected hubs. Importantly, cross-national collaborations were also frequent, particularly between U. S. institutions and their Chinese and Korean counterparts (Figure 3C). Analysis of the strongest institutional citation bursts identified the Chinese Academy of Sciences as the most influential, followed by SRM Institute of Science and Technology, Columbia University, and Baylor College of Medicine (Table 1).

**Table 1:** Top 20 institutions with the strongest citation bursts.

Institutions	Strength	Year	Begin	End
Baylor College of Medicine	10.7698	2016	2016	2019
Emory University	9.9567	2016	2016	2020
University of Texas System	9.6909	2016	2016	2020
UTMD Anderson Cancer Center	9.605	2016	2016	2020
The Institute of Automation, Chinese Academy of Sciences	20.8282	2016	2017	2019
Columbia University	10.8775	2016	2017	2021
Barrow Neurological Institute	10.7627	2016	2017	2020
Taipei Medical University Hospital	8.7109	2016	2017	2021
University of Cologne	8.7109	2016	2017	2021
Chinese Academy of Sciences	11.7169	2016	2018	2019
University of Chinese Academy of Sciences	9.7641	2016	2018	2019
Sungkyunkwan University (SKKU)	8.7801	2016	2018	2021
Chalmers University of Technology	8.4372	2016	2018	2020
University of Washington	10.158	2016	2019	2021
Xidian University	9.9609	2016	2019	2021
University of Melbourne	8.6327	2016	2020	2022
SRM Institute of Science and Technology	10.9174	2016	2023	2025
Southern Medical University	10.3596	2016	2023	2025
Vellore Institute of Technology (VIT)	8.729	2016	2023	2025

### High-impact journals and prolific authors

**Figure 4:**
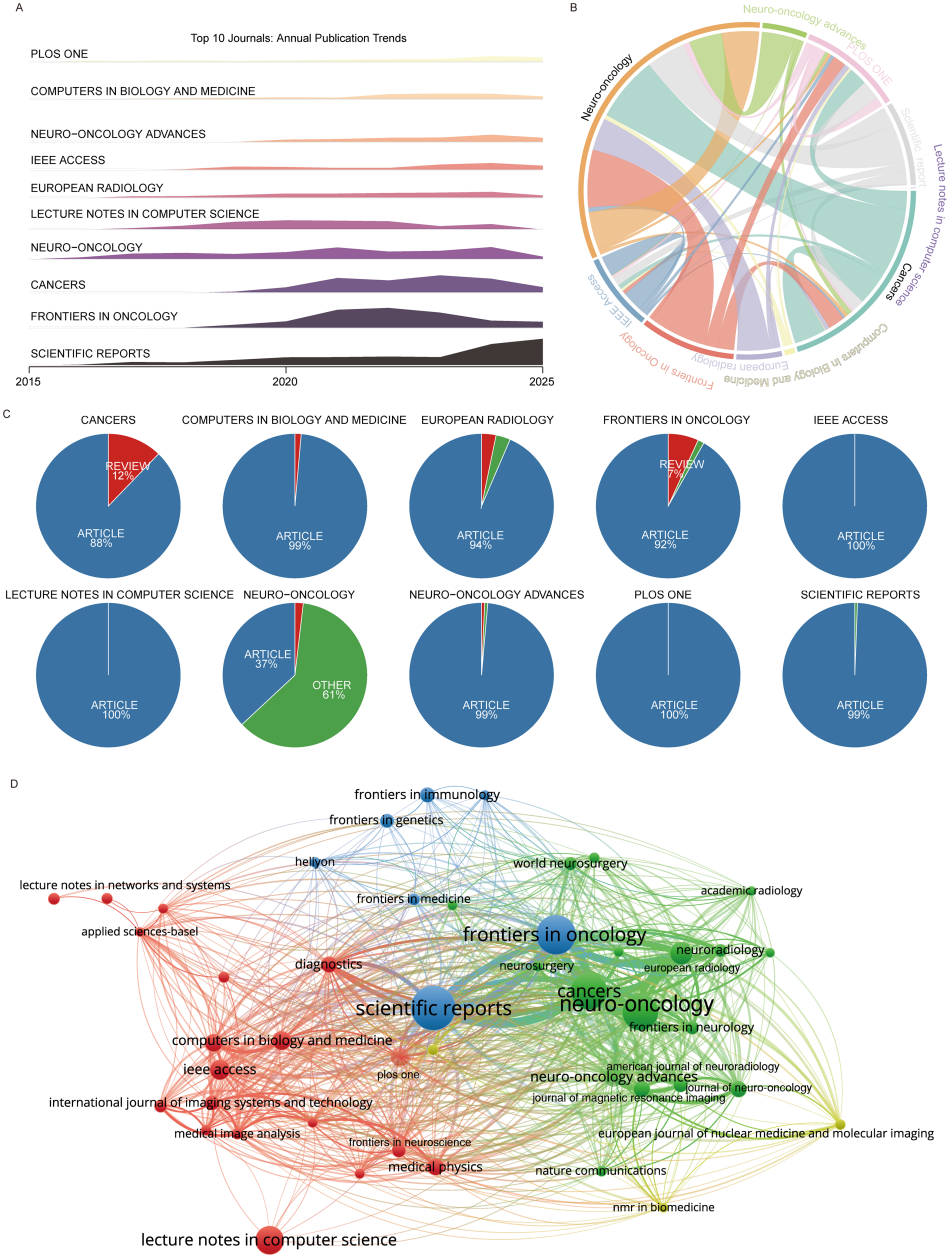
High-impact journals. **(A)** Publication trends of the top 10 journals. **(B)** Co-citation relationships among the top 10 journals. **(C)** Proportion of article vs. review publications in the top 10 journals. **(D)** Journal co-authorship and citation network.

Analysis of publication venues revealed that *Scientific Reports* was the most productive journal, publishing 511 articles that have been cited more than 10,000 times. Other high-output journals included *Frontiers in Oncology*, *Cancers*, and *Neuro-Oncology*. Journals with high citation impact included Medical Image Analysis (65 articles, 9,665 citations) and *Scientific Data* (17 articles, 4,439 citations), with the latter achieving the highest average citations per paper (Table 2). Most of the top 10 journals demonstrated an overall increasing trend in publications over time, with *Scientific Reports* showing the most pronounced growth, whereas journals such as *Frontiers in Oncology*, *Cancers*, and *Lecture Notes in Computer Science* exhibited peak activity during the mid-study period (Figure 4A).

**Table 2:** Top 20 journals with the most citations.

Journal	Documents	Citations	Citations per Doc
Scientific reports	511	10,083	19.73189824
Medical image analysis	65	9,665	148.6923077
Neuro-oncology	379	7,090	18.70712401
Frontiers in oncology	427	6,395	14.9765808
European radiology	186	6,187	33.26344086
Computers in biology and medicine	144	5,985	41.5625
Cancers	385	5,498	14.28051948
Ieee access	157	5,298	33.74522293
Ieee transactions on medical imaging	53	5,051	95.30188679
Biomedical signal processing and control	131	4,972	37.95419847
Scientific data	17	4,439	261.1176471
Journal of magnetic resonance imaging	120	4,293	35.775
Lecture notes in computer science	263	3,884	14.76806084
American journal of neuroradiology	97	3,772	38.88659794
Sensors	57	2,700	47.36842105
Nature communications	59	2,665	45.16949153
Journal of neuro-oncology	114	2,474	21.70175439
Remote sensing	66	2,460	37.27272727
Clinical cancer research	36	2,413	67.02777778
Frontiers in neuroscience	93	2,393	25.7311828

In terms of document type, the top 10 journals predominantly published research articles, with the exception of *Neuro-Oncology* and *Cancers*, where ~40% of papers were non-article formats (e.g., commentaries, case reports, and letters) (Figure 4C). Citation network analysis revealed that *Neuro-Oncology* and *Cancers* were central in the journal co-citation structure. Co-authorship analysis of journals publishing more than 25 articles showed *Scientific Reports* as a central hub, alongside *Frontiers in Oncology*, *Cancers*, and *Neuro-Oncology* (Figures 4B,D).

**Figure 5:**
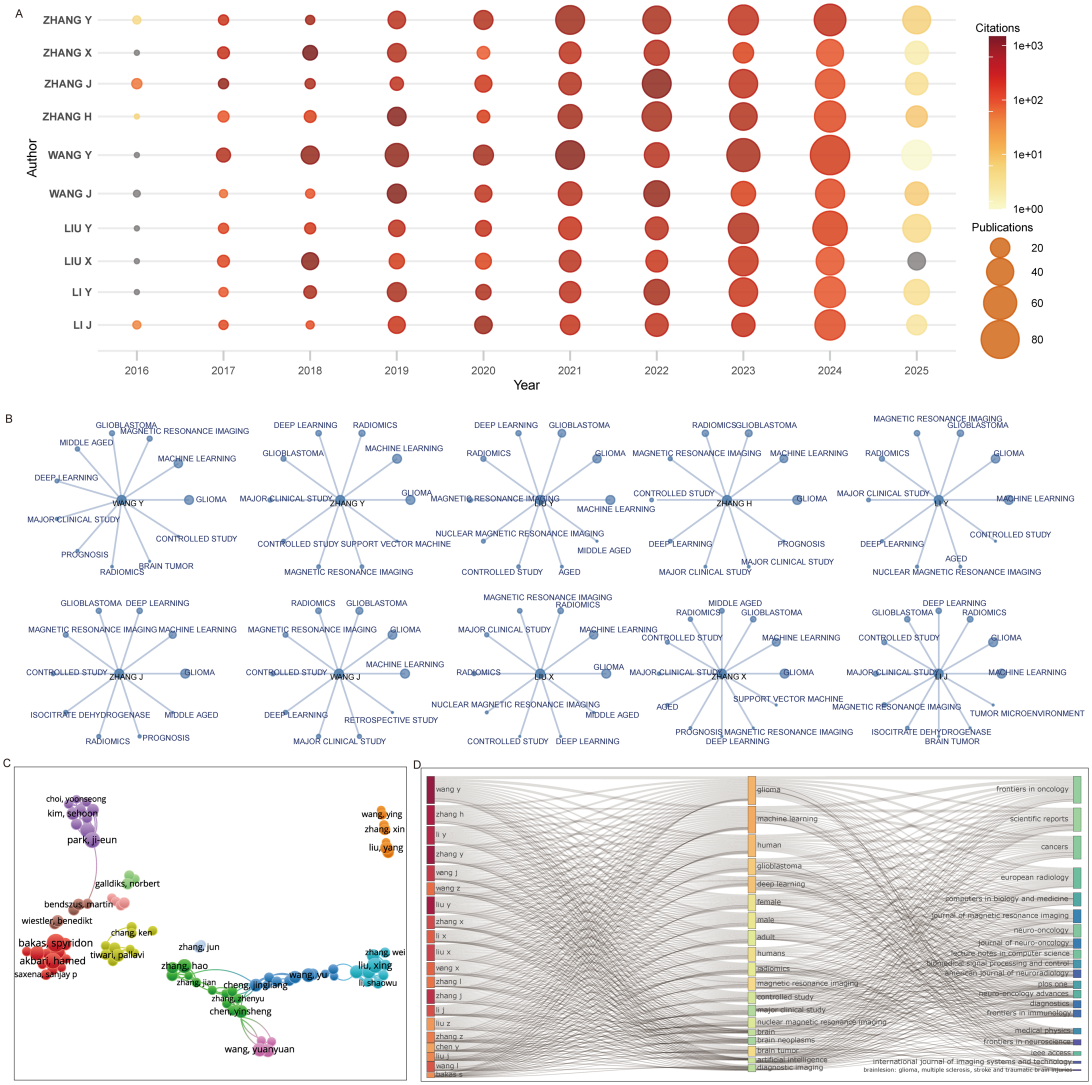
Prolific authors and their research domains. **(A)** Publication and citation trends of the top 10 authors. **(B)** High-frequency keywords used by the top 10 authors. **(C)** Co-authorship network of leading authors. **(D)** Three-field plot showing associations among authors, keywords, and journals.

The top 10 most productive authors had all been active in the field for over a decade, with nearly all reaching peak output in 2023–2024, a trend that is likely to continue into 2025 (Figure 5A). High-frequency keywords in their publications consistently included machine learning and deep learning, with nine of the 10 authors also frequently using magnetic resonance imaging (Figure 5B). Collaboration network analysis revealed several author clusters: one large hub centered around Zhang Hao, Zhang Jian, and Chen Yinsheng; another led by Kim Sehoon and Park Ji-eun; and a third centered around Bakas Spyridon and Akbari Hamed (Figure 5C). Machine learning, deep learning, radiomics, and magnetic resonance imaging were common themes across the work of the top 20 authors, and their outputs were published primarily in the top 20 identified journals (Figure 5D).

### Research hotspots and their temporal evolution

**Figure 6:**
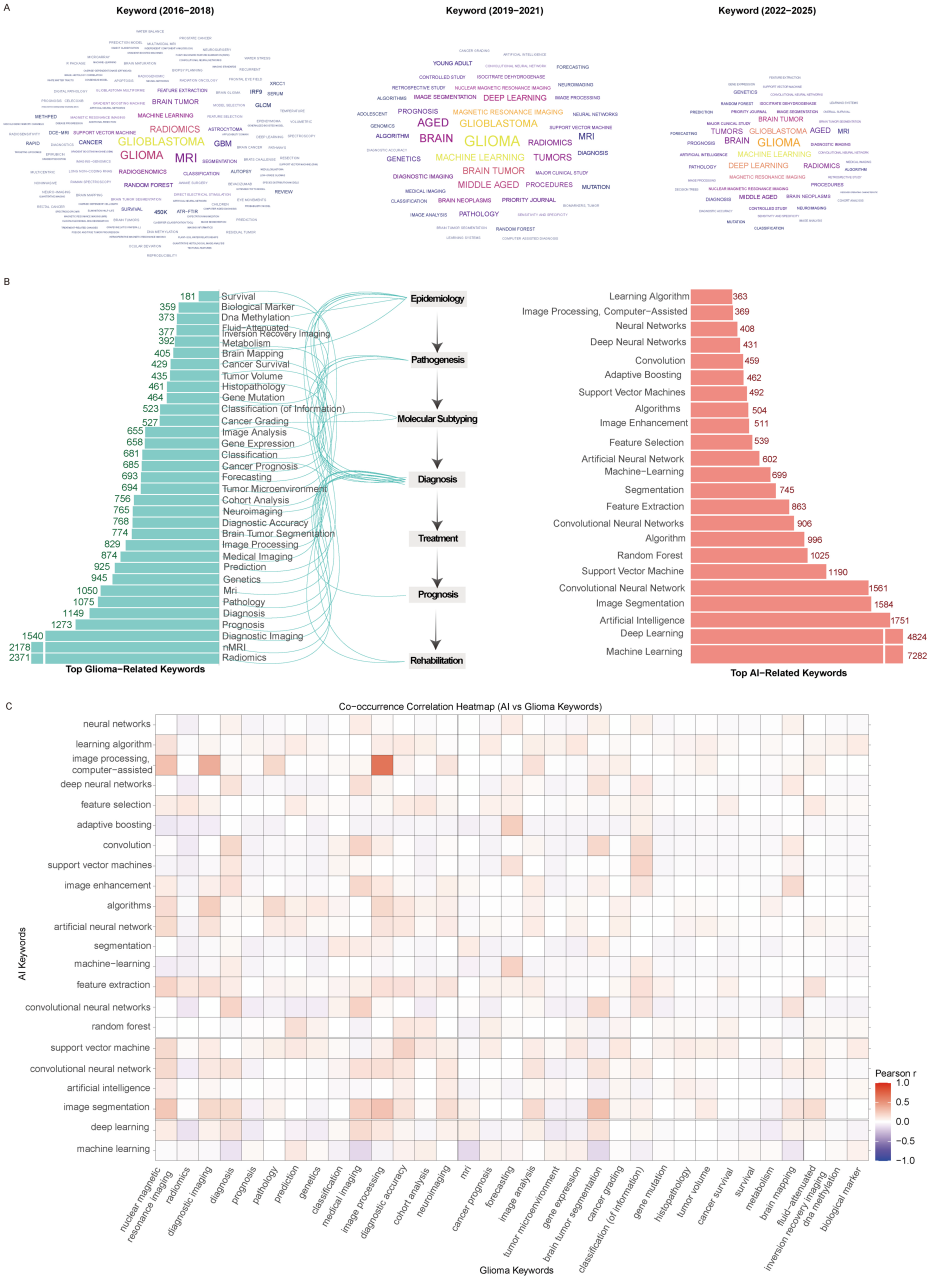
Research hotspots. **(A)** Keyword clouds across different time periods. **(B)** Top glioma- and AI-related keywords. **(C)** Correlation network between glioma and AI-related keywords.

Keyword analysis revealed distinct temporal patterns in the emergence and development of research hotspots. According to the keyword cloud stratified by time, machine learning first appeared during 2016–2018 and subsequently gained increasing prominence, whereas deep learning emerged slightly later (2019–2021), but rapidly reached comparable prevalence with machine learning by 2022–2025. Throughout the entire study period, imaging-related terms such as MRI, radiomics, and image segmentation remained consistently active (Figure 6A).

Similar patterns were observed in the most frequently occurring glioma-related keywords. Terms including radiomics, nMRI, diagnostic imaging, MRI, and medical imaging each occurred more than 750 times, underscoring the dominance of imaging-related research (Figure 6B). Other frequently appearing keywords reflected diverse thematic directions: epidemiology-related (gene mutation, gene expression), pathogenesis-related (tumor microenvironment, molecular subtyping, cancer grading), diagnostic (biological markers, image analysis), and rehabilitation (cancer survival). Notably, no treatment-related keywords appeared in the high-frequency list. Among AI-related keywords, the top five were machine learning, deep learning, artificial intelligence, image segmentation, and convolutional neural networks, each with >1,500 occurrences (Figure 6B). Correlation analysis using Pearson’s r highlighted strong associations between AI-related terms and glioma-related keywords. Imaging terms such as nuclear magnetic resonance imaging and diagnostic imaging demonstrated particularly close associations with AI concepts including feature selection, artificial neural networks, feature extraction, and image segmentation. Several AI methods (deep neural networks, support vector machines, convolutional networks, image enhancement) showed strong correlations with diagnosis-related terms, while prognosis-related terms were associated with algorithms and learning models but with weaker correlation strengths. Interestingly, classification-related keywords also demonstrated strong associations with multiple AI terms. Additional links were observed between AI concepts and glioma-related terms such as brain mapping and fluid-attenuated inversion recovery imaging (Figure 6C).

**Figure 7:**
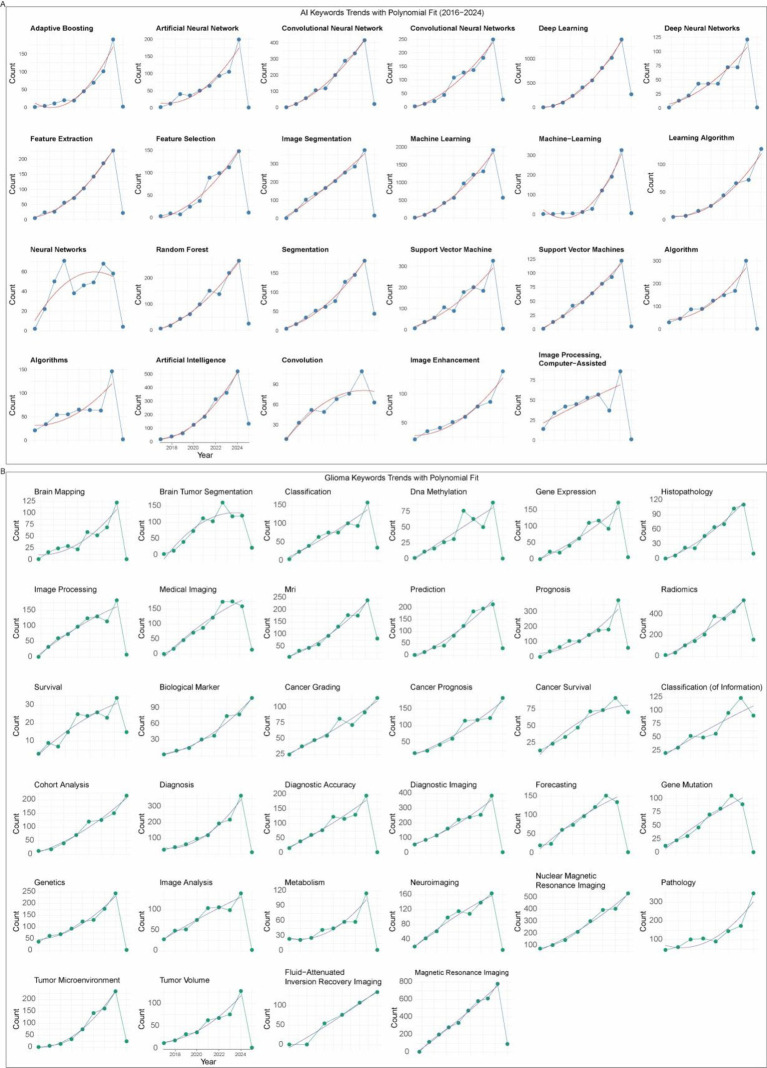
Temporal evolution of research hotspots. **(A)** Trends and quadratic fits of AI-related keywords. **(B)** Trends and quadratic fits of glioma-related keywords.

We further explored the temporal dynamics of AI- and glioma-related keywords. Both groups showed rapid growth after 2016, with most keywords fitting a quadratic growth model (*R*^2^ > 0.9), except for a few such as neural networks and algorithms (Figures 7A,B). This suggests that research interest in both AI methodologies and glioma-specific topics has expanded synergistically and continues to accelerate.

Our analysis of the top 20 references with the strongest citation bursts revealed that research in this domain has been predominantly driven by studies focusing on medical image analysis. Fourteen out of the 20 highly cited works addressed core tasks in image processing, including image segmentation, feature extraction, and pattern identification, reflecting the central role of imaging-based AI approaches in glioma research. Beyond imaging, one study was related to natural language models, highlighting the potential of text-mining and language-driven AI for knowledge discovery. Another article focused on genomic analysis, suggesting an emerging cross-disciplinary link between molecular profiling and AI-based prediction. Additionally, three highly cited publications were clinical guidelines for glioma diagnosis and treatment, underlining the critical role of standardized clinical frameworks in guiding research and translation. Collectively, these findings indicate that while medical imaging remains the dominant driver of citation impact, complementary directions such as genomics, natural language processing, and clinical practice guidelines are gaining visibility, signaling a trend toward more integrative research landscape (Table 3).

**Table 3:** Top 20 references with the strongest citation bursts.

References	Information	Year	Strength	Begin	End	2016–2025
The Multimodal Brain Tumor Image Segmentation Benchmark (BRATS)	Menze BH, 2015, IEEE T MED IMAGING, V34, P1993, DOI: 10.1109/TMI.2014.2377694	2015	118.42	2016	2020	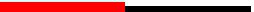
Decoding tumour phenotype by noninvasive imaging using a quantitative radiomics approach	Aerts HJWL, 2014, NAT COMMUN, V5, P0, DOI: 10.1038/ncomms5006	2014	60.61	2016	2019	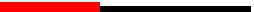
Radiomics: Images Are More than Pictures, They Are Data	Gillies RJ, 2016, RADIOLOGY, V278, P563, DOI: 10.1148/radiol.2015151169	2016	60.03	2016	2021	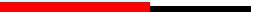
Comprehensive, Integrative Genomic Analysis of Diffuse Lower-Grade Gliomas	Brat DJ, 2015, NEW ENGL J MED, V372, P2481, DOI: 10.1056/NEJMoa1402121	2015	38.28	2016	2020	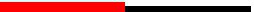
Glioblastoma Multiforme: Exploratory Radiogenomic Analysis by Using Quantitative Image Features	Gevaert O, 2014, RADIOLOGY, V273, P168, DOI: 10.1148/radiol.14131731	2014	31.24	2016	2019	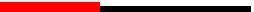
MR Imaging Predictors of Molecular Profile and Survival: Multi-institutional Study of the TCGA Glioblastoma Data Set	Gutman DA, 2013, RADIOLOGY, V267, P560, DOI: 10.1148/radiol.13120118	2013	29.24	2016	2018	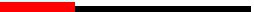
Magnetic resonance image features identify glioblastoma phenotypic subtypes with distinct molecular pathway activities	Itakura H, 2015, SCI TRANSL MED, V7, P0, DOI: 10.1126/scitranslmed.aaa7582	2015	26.4	2016	2020	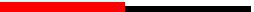
Brain Tumor Segmentation Using Convolutional Neural Networks in MRI Images	Pereira S, 2016, IEEE T MED IMAGING, V35, P1240, DOI: 10.1109/TMI.2016.2538465	2016	38.11	2017	2021	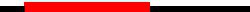
Radiomic Profiling of Glioblastoma: Identifying an Imaging Predictor of Patient Survival with Improved Performance over Established Clinical and Radiologic Risk Models	Kickingereder P, 2016, RADIOLOGY, V280, P880, DOI: 10.1148/radiol.2016160845	2016	30.53	2017	2020	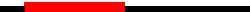
Imaging patterns predict patient survival and molecular subtype in glioblastoma via machine learning techniques	Macyszyn L, 2016, NEURO-ONCOLOGY, V18, P417, DOI: 10.1093/neuonc/nov127	2016	26.59	2017	2021	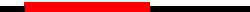
The 2016 World Health Organization Classification of Tumors of the Central Nervous System: a summary	Louis DN, 2016, ACTA NEUROPATHOL, V131, P803, DOI: 10.1007/s00401-016-1545-1	2016	106.19	2018	2021	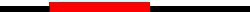
U-Net: Convolutional Networks for Biomedical Image Segmentation	Ronneberger O, 2015, LECT NOTES COMPUT SC, V9351, P234, DOI: 10.1007/978-3-319-24574-4_28	2015	52.35	2018	2020	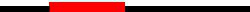
Efficient multi-scale 3D CNN with fully connected CRF for accurate brain lesion segmentation	Kamnitsas K, 2017, MED IMAGE ANAL, V36, P61, DOI: 10.1016/j.media.2016.10.004	2017	24.29	2018	2020	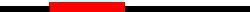
Advancing The Cancer Genome Atlas glioma MRI collections with expert segmentation labels and radiomic features	Bakas S, 2017, SCI DATA, V4, P0, DOI: 10.1038/sdata.2017.117	2017	55.89	2019	2022	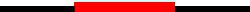
Deep Residual Learning for Image Recognition	He KM, 2016, PROC CVPR IEEE, V0, PP770, DOI: 10.1109/CVPR.2016.90	2016	31.12	2019	2021	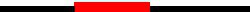
Computational Radiomics System to Decode the Radiographic Phenotype	van Griethuysen JJM, 2017, CANCER RES, V77, PE104, DOI: 10.1158/0008-5472.CAN-17-0339	2017	32.34	2020	2022	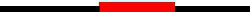
SEDE-GPS: socio-economic data enrichment based on GPS information	Sperlea T, 2018, BMC BIOINFORMATICS, V19, P0, DOI: 10.1186/s12859-018-2419-4	2018	27.18	2022	2023	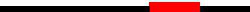
Introducing v0.5 of the AI Safety Benchmark from MLCommons	Baid U, 2021, ARXIV, V0, P0	2021	25.57	2022	2025	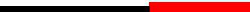
The 2021 WHO Classification of Tumors of the Central Nervous System: a summary	Louis DN, 2021, NEURO-ONCOLOGY, V23, P1231, DOI: 10.1093/neuonc/noab106	2021	147.35	2023	2025	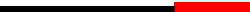
EANO guidelines on the diagnosis and treatment of diffuse gliomas of adulthood	Weller M, 2021, NAT REV CLIN ONCOL, V18, P170, DOI: 10.1038/s41571-020-00447-z	2021	23.73	2023	2025	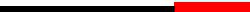

## Discussion

In this bibliometric study, we systematically explored the application of AI in the field of glioma research from January 2016 to June 2025 by analyzing publications retrieved from PubMed, Web of Science, and Scopus. Using a combination of CiteSpace, VOSviewer, and Bibliometrix, we mapped the scientific landscape, evaluated the contributions of countries, institutions, journals, and authors, and identified research hotspots and their temporal evolution. This comprehensive overview provides a quantitative and qualitative framework to understand how AI has been increasingly integrated into glioma research and where future directions may lie. Our analysis corroborates and extends findings from prior scientometric works in neuro-oncology by specifically quantifying the AI-centric shift, confirming the pivotal role of deep learning after 2016, and revealing the distinct yet complementary research trajectories of major contributing nations like the U. S. and China.

At the country and institutional level, the dominance of the U. S. and China reflects their substantial investment in AI and biomedical research. The U. S. shows a long-standing leadership with strong citation bursts, indicative of foundational and widely influential contributions. In contrast, China has demonstrated an explosive increase in publication volume in recent years, highlighting rapid capacity building and research expansion. Notably, India has exhibited a rapidly growing publication trend, which may be attributed to the rapid development of its higher education system and substantial national support in recent years. Meanwhile, although South Korea’s publication volume does not rank among the highest, its citation performance is exceptional. This could be explained by the presence of several highly influential research groups in the country that produce systematic and impactful work, resulting in research outputs that achieve significant academic influence despite a smaller quantitative footprint. Interestingly, institutions tend to cluster regionally, with Chinese universities forming domestic networks, while U. S. and Canadian institutions create tightly connected hubs across North America. Cross-continental collaborations are emerging, particularly between North America and Asia, suggesting that future breakthroughs may increasingly rely on international, interdisciplinary partnerships. Journals such as *Scientific Reports*, *Frontiers in Oncology*, and *Neuro-Oncology* provide major platforms for disseminating findings, while high-impact contributions often appear in specialized venues like Medical Image Analysis. These patterns indicate that the field has a dual publication trajectory: high-volume generalist outlets for dissemination and specialized high-impact journals for methodological advances. Similarly, the most prolific authors are distributed across multiple continents, and their networks show the field’s reliance on collaborative hubs rather than isolated researchers.

From a thematic perspective, the co-evolution of AI-related and glioma-related keywords underscores the growing synergy between computational techniques and cancer treating (21, 22). Early research was dominated by machine learning, followed by the emergence of deep learning as a transformative methodology. In terms of frequency, “machine learning” and “deep learning” are the most prominent AI-related keywords. Methodologically, deep learning-as a subset of machine learning-has further eliminated the need for manual feature engineering by employing multi-layer neural networks that mimic the structure of the human brain (23). Deep learning shows strong positive correlations with several imaging-related keywords such as “medical imaging,” “tumor segmentation,” and “MRI,” indicating its primary involvement in tumor diagnosis and classification. This is further supported by the close association between “image processing, computer-assisted” and terms like “MRI,” “diagnostic imaging,” “medical imaging,” and “image analysis.” Additionally, MRI-related AI keywords include “convolutional neural network,” “support vector machine,” and “algorithm,” a pattern also observed in “image processing” and “medical imaging,” collectively reflecting diverse efforts to apply AI to the analysis of tumor-related medical images. Several AI algorithms have demonstrated sensitivity and accuracy comparable to human experts in this field (24).

Of note, several keywords related to epidemiology and pathogenesis also appear among the top keywords, such as histopathology, gene mutation, gene expression, forecasting, and genetics. This suggests an extension of AI into these domains (25). For instance, “genetics” shows a certain positive correlation with AI keywords like “algorithms,” “random forest,” and “support vector machine.” Similarly, “gene mutation” exhibits comparable patterns, being associated with “feature extraction” and “support vector machine.” Compared to image processing, different types of cancer share certain fixed patterns and common features in medical imaging, making AI applications in this area relatively more straightforward-as reflected in our analysis, where this field has advanced more rapidly. In contrast, epidemiology and pathogenesis exhibit high heterogeneity not only across different cancers but also among individual patients (26, 27). This is particularly true at the genetic level, where knowledge itself remains in the exploratory stage. Taken together, AI applications in these areas are still under active investigation but face greater challenges due to biological complexity and data variability.

Keywords related to prognosis and rehabilitation, such as “cancer prognosis,” “cancer survival,” and “survival,” have also been observed, indicating the permeation of AI into these clinical areas (28, 29). “Cancer prognosis” is positively correlated with keywords such as “learning algorithm,” “feature selection,” “random forest,” and “support vector machine,” while “cancer survival” is mainly linked to “feature selection,” “random forest,” and “support vector machine.” This suggests that AI is increasingly being utilized to identify prognostic biomarkers and build predictive models for survival outcomes (30, 31), though the methodologies remain largely reliant on classical machine learning approaches for feature interpretation and model transparency.

The relative scarcity of treatment-related keywords suggests that AI applications in glioma therapeutics-such as treatment planning, prediction of therapeutic response, or integration with novel modalities like immunotherapy-remain less explored (32). This disparity likely stems from the greater accessibility and standardization of imaging data compared to the complex, heterogeneous, and longitudinal data required for modeling treatment outcomes.

Looking forward, the strong correlation patterns observed in this study point toward several promising research frontiers. First, the well-established linkage between AI and diagnostic imaging suggests that multimodal imaging integration-combining structural MRI, functional sequences, PET, radiomics, and radiogenomics-will become increasingly important. Such integration is expected to enable more accurate tumor grading, molecular subtyping, and prognosis prediction (33, 34). Second, the association between AI methods and molecular/epidemiological terms implies a growing role for integrative multi-omics approaches, where AI could uncover latent patterns linking genomic mutations, transcriptomic profiles, and imaging phenotypes. This would facilitate the development of more personalized diagnostic and prognostic models that account for both morphological and molecular characteristics of gliomas.

Third, the underrepresentation of treatment-related terms highlights a significant unmet need and opportunity. AI has enormous potential to optimize surgical planning through improved tumor boundary delineation, enhance radiotherapy targeting via dose optimization algorithms, and predict individual patient responses to various therapeutic regimens. However, these applications remain at an early stage of development. Future research should focus on creating standardized frameworks for collecting and sharing high-quality clinical treatment data, including surgical outcomes, radiation therapy parameters, and longitudinal treatment responses. Additionally, the development of specialized AI architectures capable of handling the temporal nature of treatment data and the complex relationships between different treatment modalities will be crucial for advancing this field.

Finally, with the rapid development of large language models (LLMs) and foundation models, natural language processing applied to electronic health records, pathology reports, and clinical trial data may provide a complementary dimension that bridges clinical practice and research discovery (21). These models could help extract valuable insights from unstructured clinical text, generate synthetic data for rare glioma subtypes, and facilitate knowledge discovery from the vast corpus of existing medical literature. The integration of these diverse data types through advanced AI approaches promises to create a more comprehensive understanding of glioma biology and treatment, ultimately leading to improved patient outcomes.

In summary, this bibliometric study demonstrates that AI applications in glioma research have grown rapidly since 2016, with imaging-centered approaches dominating the landscape and deep learning driving the methodological surge. High-impact contributions are concentrated in a limited number of countries, institutions, and journals, yet collaborative networks are expanding globally. Research hotspots reveal a clear emphasis on diagnosis and classification, while underdeveloped areas such as treatment optimization, prognostic modeling, and multimodal integration represent future directions. As AI technologies continue to mature, their successful translation into glioma care will depend on interdisciplinary collaborations that unite data scientists, neuroscientists, and clinicians, ultimately aiming to improve patient outcomes in this challenging disease.

## Data Availability

The original contributions presented in the study are included in the article/Supplementary material, further inquiries can be directed to the corresponding author.

## References

[1] Weller M van den Bent M Preusser M Le Rhun E Tonn JC Minniti G . EANO guidelines on the diagnosis and treatment of diffuse gliomas of adulthood. Nat Rev Clin Oncol. (2021) 18:170–86. doi: 10.1038/s41571-020-00447-z, 33293629 PMC7904519

[2] Louis DN Perry A Wesseling P Brat DJ Cree IA Figarella-Branger D . The 2021 WHO classification of tumors of the central nervous system: a summary. Neuro-Oncology. (2021) 23:1231–51. doi: 10.1093/neuonc/noab106, 34185076 PMC8328013

[3] Jiang T Tang GF Lin Y Peng XX Zhang X Zhai XW . Prevalence estimates for primary brain tumors in China: a multi-center cross-sectional study. Chin Med J. (2011) 124:2578–83. doi: 10.3760/cma.j.issn.0366-6999.2011.17.003 22040406

[4] Miller AM Shah RH Pentsova EI Pourmaleki M Briggs S Distefano N . Tracking tumour evolution in glioma through liquid biopsies of cerebrospinal fluid. Nature. (2019) 565:654–8. doi: 10.1038/s41586-019-0882-3, 30675060 PMC6457907

[5] Baek C Laurenge A Touat M. Advances in the treatment of IDH-mutant gliomas. Curr Opin Neurol. (2024) 37:708–16. doi: 10.1097/WCO.0000000000001316, 39253756

[6] Eyupoglu IY Buchfelder M Savaskan NE. Surgical resection of malignant gliomas-role in optimizing patient outcome. Nat Rev Neurol. (2013) 9:141–51. doi: 10.1038/nrneurol.2012.279 23358480

[7] Yasinjan F Xing Y Geng H Guo R Yang L Liu Z . Immunotherapy: a promising approach for glioma treatment. Front Immunol. (2023) 14:1255611. doi: 10.3389/fimmu.2023.1255611, 37744349 PMC10512462

[8] Piil K Jarden M Jakobsen J Christensen KB Juhler M. A longitudinal, qualitative and quantitative exploration of daily life and need for rehabilitation among patients with high-grade gliomas and their caregivers. BMJ Open. (2013) 3:e003183. doi: 10.1136/bmjopen-2013-003183, 23847270 PMC3710984

[9] Hollon T Jiang C Chowdury A Nasir-Moin M Kondepudi A Aabedi A . Artificial-intelligence-based molecular classification of diffuse gliomas using rapid, label-free optical imaging. Nat Med. (2023) 29:828–32. doi: 10.1038/s41591-023-02252-4, 36959422 PMC10445531

[10] Nakhate V Gonzalez Castro LN. Artificial intelligence in neuro-oncology. Front Neurosci. (2023) 17:1217629. doi: 10.3389/fnins.2023.1217629, 38161802 PMC10755952

[11] Tak D Garomsa BA Zapaishchykova A Ye Z Vajapeyam S Mahootiha M . Longitudinal risk prediction for pediatric glioma with temporal deep learning. NEJM AI. (2025) 2. doi: 10.1101/2024.06.04.24308434 PMC12176428 40535328

[12] Verma R Alban TJ Parthasarathy P Mokhtari M Toro Castano P Cohen ML . Sexually dimorphic computational histopathological signatures prognostic of overall survival in high-grade gliomas via deep learning. Sci Adv. (2024) 10:eadi0302. doi: 10.1126/sciadv.adi0302, 39178259 PMC11343024

[13] Nakagaki R Debsarkar SS Kawanaka H Aronow BJ Prasath VBS. Deep learning-based IDH1 gene mutation prediction using histopathological imaging and clinical data. Comput Biol Med. (2024) 179:108902. doi: 10.1016/j.compbiomed.2024.108902, 39038392 PMC13215039

[14] Liu Y Zhong R Li YX Yu C Zhang J Kou Z. Mapping the evolution of kainate receptor research over five decades: trends, hotspots, and emerging frontiers. Naunyn Schmiedeberg's Arch Pharmacol. (2025). doi: 10.1007/s00210-025-04540-x 40848135

[15] Liu Y Jia Y Kou Z. Bibliometric analysis of NMDA receptors: 2015-2024. Front Pharmacol. (2025) 16:1614831. doi: 10.3389/fphar.2025.1614831, 40548058 PMC12179074

[16] Liu Y Kou Z. Therapeutic shifts and scientific influence in treatment-resistant depression research: a data-driven perspective. Biol Psychiatry Global Open Sci. (2026) 6:100610. doi: 10.1016/j.bpsgos.2025.100610, 41141385 PMC12552951

[17] Liu Y Zhong R Wu X Zhang J Kou Z. Global hotspots and trends in AMPA receptor research (2000-2025): a bibliometric and visualization analysis. Naunyn Schmiedeberg's Arch Pharmacol. (2025). doi: 10.1007/s00210-025-04679-7 41074965

[18] Chen C. CiteSpace II: detecting and visualizing emerging trends and transient patterns in scientific literature. J Am Soc Inf Sci Technol. (2005) 57:359–77. doi: 10.1002/asi.20317

[19] van Eck NJ Waltman L Dekker R van den Berg J. A comparison of two techniques for bibliometric mapping: multidimensional scaling and VOS. J Am Soc Inf Sci Technol. (2010) 61:2405–16. doi: 10.1002/asi.21421

[20] Aria M Cuccurullo C. Bibliometrix: an R-tool for comprehensive science mapping analysis. J Informetr. (2017) 11:959–75. doi: 10.1016/j.joi.2017.08.007

[21] Fahrner LJ Chen E Topol E Rajpurkar P. The generative era of medical AI. Cell. (2025) 188:3648–60. doi: 10.1016/j.cell.2025.05.018, 40645169

[22] Perez-Lopez R Ghaffari Laleh N Mahmood F Kather JN. A guide to artificial intelligence for cancer researchers. Nat Rev Cancer. (2024) 24:427–41. doi: 10.1038/s41568-024-00694-7, 38755439

[23] Tran KA Kondrashova O Bradley A Williams ED Pearson JV Waddell N. Deep learning in cancer diagnosis, prognosis and treatment selection. Genome Med. (2021) 13:152. doi: 10.1186/s13073-021-00968-x, 34579788 PMC8477474

[24] Lv C Shu XJ Qiu J Xiong ZC Bo Ye J Bo Li S . AI-enabled precise brain tumor segmentation by integrating Refinenet and contour-constrained features in MRI images. Med Phys. (2025) 52:e17958. doi: 10.1002/mp.17958, 40660802

[25] Moon HH Jeong J Park JE Kim N Choi C Kim YH . Generative AI in glioma: ensuring diversity in training image phenotypes to improve diagnostic performance for IDH mutation prediction. Neuro-Oncology. (2024) 26:1124–35. doi: 10.1093/neuonc/noae012, 38253989 PMC11145451

[26] Blanco-Carmona E Narayanan A Hernandez I Nieto JC Elosua-Bayes M Sun X . Tumor heterogeneity and tumor-microglia interactions in primary and recurrent IDH1-mutant gliomas. Cell Rep Med. (2023) 4:101249. doi: 10.1016/j.xcrm.2023.101249 37883975 PMC10694621

[27] Lhermitte B Wolf T Chenard MP Coca A Todeschi J Proust F . Molecular heterogeneity in BRAF-mutant gliomas: diagnostic, prognostic, and therapeutic implications. Cancer. (2023) 15. doi: 10.3390/cancers15041268, 36831610 PMC9954401

[28] Zhang B Shi H Wang H. Machine learning and AI in cancer prognosis, prediction, and treatment selection: a critical approach. J Multidiscip Healthc. (2023) 16:1779–91. doi: 10.2147/JMDH.S410301 37398894 PMC10312208

[29] Rintala TJ Napolitano F Fortino V. Multi-task deep latent spaces for cancer survival and drug sensitivity prediction. Bioinformatics. (2024) 40:ii182–9. doi: 10.1093/bioinformatics/btae388, 39230696 PMC11520233

[30] Tambi R Zehra B Vijayakumar A Satsangi D Uddin M Berdiev BK. Artificial intelligence and omics in malignant gliomas. Physiol Genomics. (2024) 56:876–95. doi: 10.1152/physiolgenomics.00011.2024, 39437552

[31] Pasquini L Napolitano A Lucignani M Tagliente E Dellepiane F Rossi-Espagnet MC . AI and high-grade glioma for diagnosis and outcome prediction: do all machine learning models perform equally well? Front Oncol. (2021) 11:601425. doi: 10.3389/fonc.2021.601425, 34888226 PMC8649764

[32] Khalighi S Reddy K Midya A Pandav KB Madabhushi A Abedalthagafi M. Artificial intelligence in neuro-oncology: advances and challenges in brain tumor diagnosis, prognosis, and precision treatment. NPJ Precis Oncol. (2024) 8:80. doi: 10.1038/s41698-024-00575-0, 38553633 PMC10980741

[33] Bhinder B Gilvary C Madhukar NS Elemento O. Artificial intelligence in Cancer research and precision medicine. Cancer Discov. (2021) 11:900–15. doi: 10.1158/2159-8290.CD-21-0090, 33811123 PMC8034385

[34] Tiwari A Mishra S Kuo TR. Current AI technologies in cancer diagnostics and treatment. Mol Cancer. (2025) 24:159. doi: 10.1186/s12943-025-02369-9, 40457408 PMC12128506

