# Supplementary material for: Artificial intelligence in glioma research: a bibliometric analysis of global trends, hotspots, and future directions
Source: Front Neurol. 2026 Jan 12;16:1701499. doi: 10.3389/fneur.2025.1701499 (PMC12832372; doi:10.3389/fneur.2025.1701499)
Supplement: Supplementary file 1 [file Image_1.PDF]

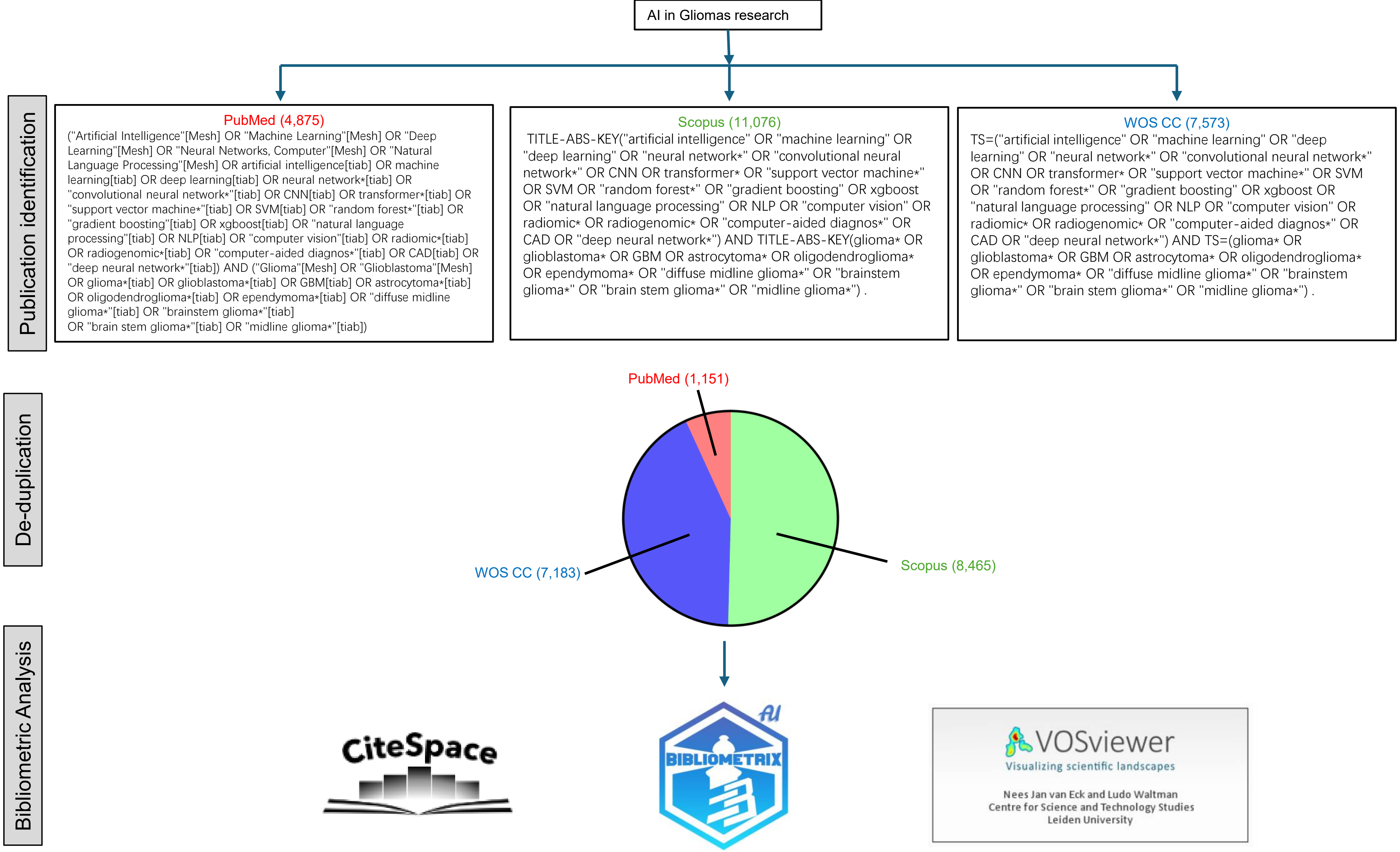

Supplementary Figure 1: Search strategies, publication de-duplication, and bibliometric analysis.
